# Is Feto-Maternal Transfusion after Cesarean Delivery Different in Singleton and Twin Pregnancy?

**DOI:** 10.3390/jcm13123609

**Published:** 2024-06-20

**Authors:** Anna Stachurska-Skrodzka, Damian Mielecki, Anna Fijałkowska, Kinga Żebrowska, Monika Kasperczak, Katarzyna Kosińska-Kaczyńska

**Affiliations:** 1 Department of Cell Biology and Immunology, Center of Postgraduate Medical Education, 01-813 Warsaw, Poland; astachurska@cmkp.edu.pl (A.S.-S.); anna.fijalkowska@cmkp.edu.pl (A.F.); 2 Department of Neurochemistry, Mossakowski Medical Research Institute, Polish Academy of Sciences, 02-106 Warsaw, Poland; dmielecki@imdik.pan.pl; 3 Department of Obstetrics, Perinatology and Neonatology, Center of Postgraduate Medical Education, 01-813 Warsaw, Poland; kinga.zebrowska@gmail.com (K.Ż); monika.kasperczak@icloud.com (M.K.)

**Keywords:** feto-maternal transfusion, cytometry, microscopy, twin pregnancy, cesarean delivery, chorionicity

## Abstract

**Background:** The aim of the study was to investigate if feto-maternal transfusion was related to the size of the fetal-maternal interface, and, therefore, was larger in twin pregnancy in comparison with singleton pregnancy. **Methods**: Blood samples from women with singleton (*n* = 11), and monochorionic (*n* = 11) and dichorionic (*n* = 13) twin gestations were tested. Flow cytometry tests with hemoglobin F, glycophorin A, and hemoglobin F and carbonic anhydrase simultaneous staining were used to detect fetal red blood cells and maternal F cells. **Results**: In all cases, the volume of feto-maternal transfusion was estimated to be low. The highest rate of fetal red blood cells in the maternal circulation was observed in the blood of women with dichorionic twin gestations both before and after delivery. An increase in fetal red blood cells was observed after cesarean section in singletons and twins. The median rate of maternal F cells was 2.23% in singleton, 2.1% in monochorionic and 3.95% in dichorionic pregnancy. **Conclusions**: Feto-maternal transfusion during pregnancy may be related to the multiplicity and chorionicity of pregnancy.

## 1. Introduction

Feto-maternal transfusion (FMT), also known as transplacental hemorrhage, is defined as the fetal red blood cell transfer into the maternal circulation throughout pregnancy and delivery. In normal pregnancy, small, clinically insignificant amounts of fetal blood can pass into the maternal circulation throughout pregnancy. During labor and delivery, an increased incidence of FMT may be related to contractions, placental separation and delivery. Invasive procedures during pregnancy, like amniocentesis, chorionic villus sampling and external cephalic version can also lead to FMT due to the damage to the villi. Abdominal trauma or placental abruption can cause significant FMT. The normal placenta is a fairly tight barrier that protects against the leakage of larger amounts of fetal erythrocytes. In 1979, Mollison estimated the magnitude of FMT on a logarithmic scale against the cumulative frequency of FMH following normal delivery, also on a logarithmic scale, as follows:

- Approximately 0.1% of women have FMH ≥ 30 mL RBC.
- Approximately 0.3% of women have FMH ≥ 10 mL RBC.
- Approximately 1% of women have FMH ≥ 3 mL RBC.
- Approximately 3% of women have FMH ≥ 1 mL RBC.
- Approximately 10% of women have FMH ≥ 0.3 mL RBC.
- Approximately 30% of women have FMH ≥ 0.1 mL RBC [1].

As a result of placental barrier interruption, the fetal red blood cells can cross into the maternal circulation [2,3,4]. If the mother’s immune system is stimulated by fetal red blood cell antigens, antibodies are produced, which may lead to the development of hemolytic disease of the fetus (HDF). IgG alloimmunoglobulins cross the placenta and bind to the antigens of the fetal red cells. The red blood cells are destroyed, phagocytosed by macrophages and removed by the liver or spleen. Hemolytic disease of the fetus/newborn may have a different course depending on the degree of anemia [5,6]. In case of severe hemolysis, hydrops fetalis and fetal demise may occur. The D antigen is the strongest immunogen responsible for the occurrence of hemolytic disease of the fetus and the newborn (HDFN) [5]. However, incompatibility in other antigens may also lead to HDF. They include the antigens of the Rh system (e.g., c, E), as well as antigens of other group systems, such as the Kell, Duffy, MNS, common antigens (e.g., Co^a^, Jr^a^), as well as low-incidence antigens (e.g., Di^a^, Wr^a^) [5,6]. The post-delivery administration of anti-D immunoglobulin (anti-RhD Ig) is a recommended mode of prophylaxis against HDF due to the D antigen incompatibility [7,8]. The underestimation of FMT volume may result in ineffective prophylaxis with the dose of anti-D immunoglobulin being too low, while the overestimation of FMT may lead to unnecessary treatment with doses that are too high, which may increase the risk of blood-transmitted diseases or allergic reactions. An excessive dose of anti-RhD Ig is also cost-ineffective. The recommended doses vary according to the gestational age and type of pregnancy and differ between countries. In the United Kingdom, 500 IU of anti-D Ig is administered within 72 h following delivery [7]. In the United States of America, 300 µg is the standard dose after delivery [8]. In Poland, the standard dose is 150 µg after singleton vaginal birth and 300 µg after cesarean or twin delivery [9]. This is due to the assumed difference in FMT between singleton and twin delivery. Different methods (flow cytometry tests, the microscopic Kleihauer-Betke method, serological agglutination test, high-performance liquid chromatography) were introduced for the measurement of the rate of fetal cells in maternal circulation [10]. Few reports are available on the analysis and comparison of FMT in singleton and twin pregnancies, and their findings are inconclusive [3,4,11,12,13]. 

In the majority of published investigations, the FMT volume was calculated using the Kleihauer-Betke test, which is less accurate compared to the cytometric method and allows for only the semi-quantitative analysis of FMT [14]. Its advantage is related to the fact that it is inexpensive and may be performed in any analytical or serological laboratory with a simple light microscope. However, in the case of the prevalence of maternal F cells in pregnant women’s circulation it carries a risk of obtaining false positive results, leading to the overestimation of FMT volume [15,16]. The Kleihauer-Betke test has poor reproducibility and requires manual measurement, which may introduce bias [17]. The hemoglobin elution stage is sensitive to pH, time and temperature. The subjective interpretation of the smear and the experience of the researcher conducting the study may also affect the results obtained. The inability to distinguish between fetal erythrocytes and F cells is the main reason for receiving false results in the Kleihauer-Betke test [18]. 

As it is hypothesized that FMT is related to the size of the fetal-maternal interface, and, therefore, it is larger in twin pregnancy in comparison with singleton pregnancy, we aimed to compare FMT after cesarean delivery in singleton and twin gestation regarding chorionicity (dichorionic and monochorionic gestation).

## 2. Materials and Methods

This was a pilot study assessing FMT. Patients who were hospitalized in the Department of Obstetrics, Perinatology and Neonatology, Center of Postgraduate Medical Education, were included in this study. Our assessments of FMT were performed between December 2022 and June 2023. The inclusion criteria consisted of the following: age above 18 years old, gestational age above 34 weeks, elective cesarean delivery. The exclusion criteria were vaginal delivery, fetal demise, fetal growth restriction, placenta previa and placenta accreta spectrum, vaginal bleeding during pregnancy, placental abruption, intrauterine procedures during pregnancy or any kind of abdominal trauma. All the women were asked to give written informed consent to participate in the study.

All included women had blood samples collected 30–60 min before cesarean section (CS) and within 30–60 min after the delivery of the placenta. Venous blood samples were collected in EDTA test tubes, using aseptic venipuncture. They were stored at 2 to 8 °C and processed within 2 h of blood collection. All analyses were performed in the Department of Cell Biology and Immunology, Center of Postgraduate Medical Education. We used a hematology analyzer (Beckman Coulter A cTdif, Indianapolis, IN, USA) to prepare red blood cell mixtures imitating FMT with the known percentage of the fetal cells (0.1, 1%). The tested blood samples were analyzed against negative controls (nonlabeled samples). FMH was assessed using flow cytometry methods: hemoglobin, glycophorin, hemoglobin and anhydrase simultaneous staining methods.

The study was conducted according to the Declaration of Helsinki. The study protocol was approved by the ethics committee at the Center of Postgraduate Medical Education (no. 86/PB/2020).

### 2.1. Hemoglobin F Staining

**Figure 1:**
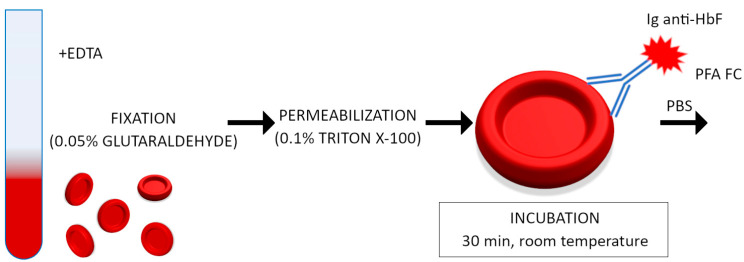
Procedure diagram of fetal hemoglobin staining.

Blood samples containing 20 µL were fixed in cold (4 °C) freshly prepared 0.05% glutaraldehyde (Sigma-Aldrich, St Louis, MI, USA) for 10 min at room temperature. After being washed 3 times in PBS buffer, the cells were permeabilized with 500 µL 0.1% Triton X-100 (Sigma-Aldrich, USA) for 10 min at room temperature and washed with PBS again. Then, red blood cells were suspended in 300 µL PBS, and 10 µL RBC suspension was incubated for 30 min at room temperature (avoiding direct light) with 5 µL of PE-conjugated anti-HbF human monoclonal antibody (BD Pharmingen, Franklin Lakes, NJ, USA) and supplemented with 70 µL PBS with 0.1% BSA. After the final wash in PBS, the cells were resuspended in 300 µL of 1% paraformaldehyde (Sigma-Aldrich, USA) and immediately acquired by flow cytometer. Erythrocyte cell samples underwent FACS analysis using FACSCanto II and FACSDiva software v8.03 (Becton Dickinson, Franklin Lakes, NJ, USA). We calibrated the flow cytometer according to the manufacturer’s instruction. A total of 50,000 events were collected in samples. The cells were gated on log forward scatter (FS) versus log side scatter (SS) dot plots to exclude debris and agglutinated RBC, then gated on SSC versus FL2 dot plots to exclude autofluorescent granulocytes. Events were collected for fluorescence signal for PE conjugated to the anti-HbF antibody with the region gated at the erythrocytes. The following groups of erythrocytes were determined: adult erythrocytes (HbF-negative), adult F cells (HbF weakly positive) and fetal erythrocytes (HbF strongly positive). The procedure of fetal hemoglobin staining is presented in Figure 1 and an example of cytometric analysis is shown in Figure S1 (Supplementary Materials).

### 2.2. Hemoglobin F (HbF) and Carbonic Anhydrase (CA) Staining 

**Figure 2:**
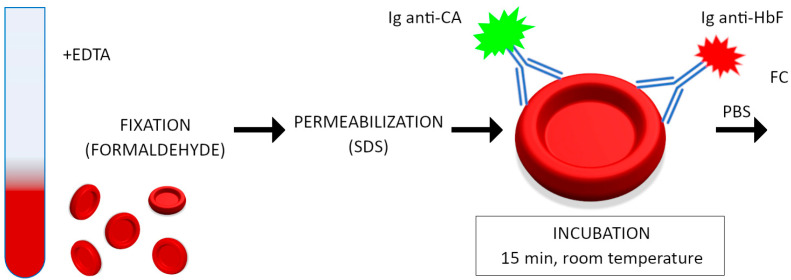
Procedure diagram of fetal hemoglobin and carbonic anhydrase staining.

In ambiguous cases of women who had F cells (with hemoglobin F), hemoglobin F and carbonic anhydrase simultaneous staining assay was additionally performed. We used the Fetal Cell Count Kit II (IQ Products, Groningen, The Netherlands) following the recommendations/procedure of the manufacturer. Briefly, 50 µL of blood samples was washed with reagent D (a rinsing solution) and the pellet was suspended in 20 µL of reagent D washing solution. Then, 100 µL of reagents A and B (fixative solutions) was added to 10 µL of RBC suspension and incubated for 30 min at room temperature. After this time, RBCs were washed with reagent D, the cell pellet was resuspended in 100 µL of reagent D and mixed with 100 µL of reagent C (a permeabilization solution). After 3 min of incubation, red blood cells were washed (reagent D), resuspended in 1 mL of reagent D and incubated with reagent E (50 µL anti-human CA-FITC polyclonal antibody) and with reagent F (50 µL anti-human HbF-PE monoclonal antibody) for 15 min at room temperature in the dark. Subsequently, the cells were washed and resuspended in reagent D (300 µL) and immediately analyzed in a flow cytometer within 30 min. Fetal RBCs were recognized by their bright HbF expression combined with a weaker CA expression. A negative control (a donor blood sample) was included and a donor blood sample with fetal erythrocytes (0.1, 1% artificial mixtures). The procedure of HbF and carbonic anhydrase staining is presented in Figure 2 and an example of the results is shown in Figure S2 (Supplementary Materials).

### 2.3. Glycophorin A (CD235a) Staining

To separate erythrocytes on cytograms, the anti-glycophorin A (a specific marker for erythrocytes only) antibody was additionally used. This allowed the elimination of other types of cells found in the blood, making the analysis more accurate. Samples (50 µL) were incubated with 1 µL of the anti-CD 235a-PE monoclonal antibody for 30 min at room temperature in the dark. Next, the stained cells were washed and immediately analyzed in a flow cytometer.

### 2.4. Statistical Analysis 

Data were described by medians and interquartile ranges or percentages, and compared using the Student’s test or the nonparametric Wilcoxon signed rank, i.e., a test used when data are not normally distributed. *p* values < 0.05 were considered statistically significant. All data preparations were performed in R 4.2.2 project and visualized with ggplot2. 

## 3. Results

### 3.1. Characteristics of the Study Group

After obtaining information about the general purpose of the study and the voluntary nature of participation, 35 women agreed and five women declined to participate in the study. A total of 35 women were included in the study: 11 with singleton, 11 with monochorionic twin and 13 with dichorionic twin pregnancy. The characteristics of the study group are presented in Table 1. Women with twin gestation delivered significantly earlier and the infants were smaller. No other differences were observed between the groups. In the whole study group, FMT was below 0.19% (the average amount was lower than 4 mL). 

### 3.2. Comparison of Fetal Red Blood Cells Diagnosed in the Maternal Circulation before Delivery in the Analyzed Groups 

**Figure 3:**
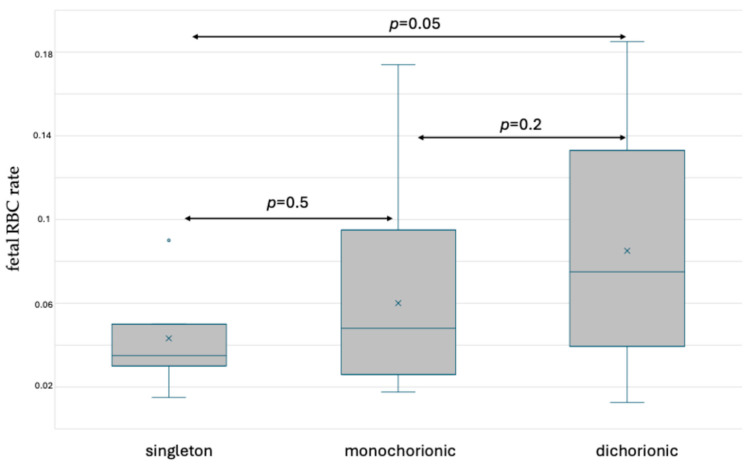
Box and whisker plots of fetal RBC ratios in singleton and twin pregnancy before delivery based on fetal hemoglobin staining. x—mean; blue line—median; blue circle—outliers.

The rates of fetal red blood cells (RBCs) in the maternal circulation before delivery were assessed using the abovementioned methods. The values measured by performing fetal hemoglobin staining and fetal hemoglobin and carbonic anhydrase simultaneous staining are presented in Table S1 (Supplementary Materials). In HbF staining, no significant differences were observed in fetal RBC ratios between the group of singleton and monochorionic gestations, or between monochorionic and dichorionic gestations. In women with dichorionic pregnancy, more fetal RBCs were found in the circulation in comparison with singleton gestations (*p* = 0.05). The results are presented in Figure 3. 

**Figure 4:**
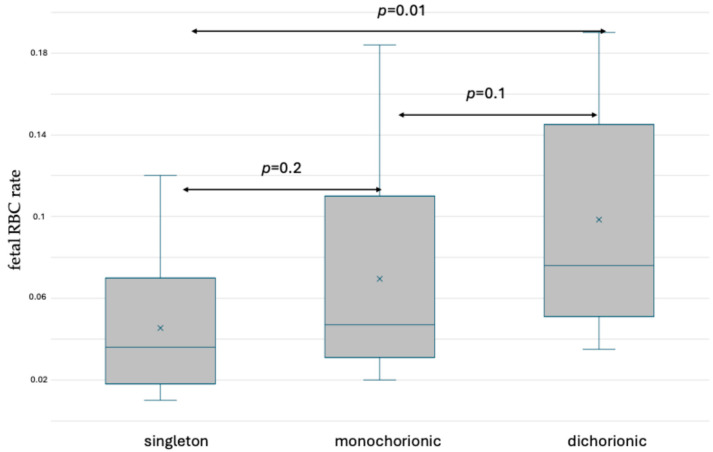
Box and whisker plots of fetal RBC ratios in singleton and twin pregnancy before delivery based on fetal hemoglobin and carbonic anhydrase staining. x—mean; blue line—median; blue circle—outliers.

The simultaneous application of anti-HbF-PE and anti-CA-FITC antibodies allowed the separation of fetal erythrocytes from maternal F cells. No significant differences were observed between singleton and monochorionic pregnancies, or between monochorionic and dichorionic pregnancies. In dichorionic gestations, the rate of fetal RBCs was significantly higher than in singleton gestations before delivery. The results are presented in Figure 4 and Table S1.

### 3.3. Comparison of Fetal RBCs Diagnosed in the Maternal Circulation before and after Delivery

**Figure 5:**
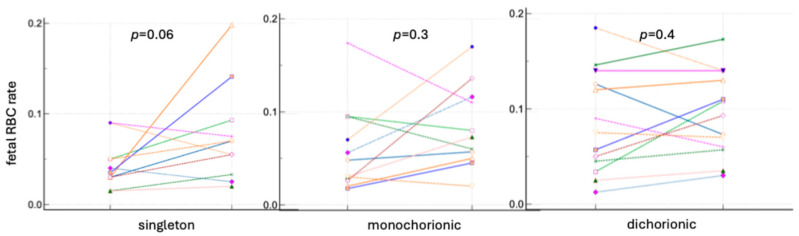
Fetal RBC ratios in singleton and twin pregnancy before and after delivery based on fetal hemoglobin staining. Lines present the fetal RBC rates before and after delivery in each patient.

**Figure 6:**
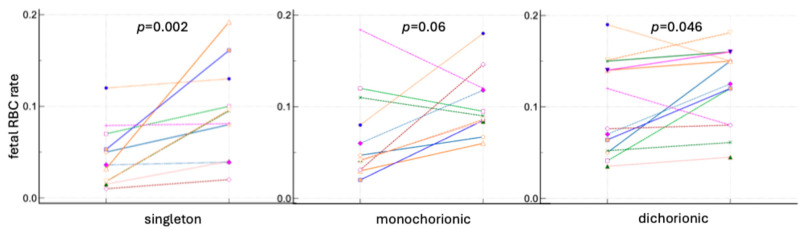
The fetal RBC ratios in singleton and twin pregnancy before and after delivery based on fetal hemoglobin and carbonic anhydrase staining. Lines present the fetal RBC rates before and after delivery in each patient.

The differences in the rates of fetal RBCs in the maternal circulation before and after delivery were analyzed. In all groups of pregnancies, an increase in the number of fetal cells was observed after cesarean section based on fetal hemoglobin and carbonic anhydrase staining. The results are presented in Figure 5 and Figure 6 and Table S1.

### 3.4. Comparison of Fetal RBCs Diagnosed in the Maternal Circulation after Delivery in the Analyzed Groups

**Figure 7:**
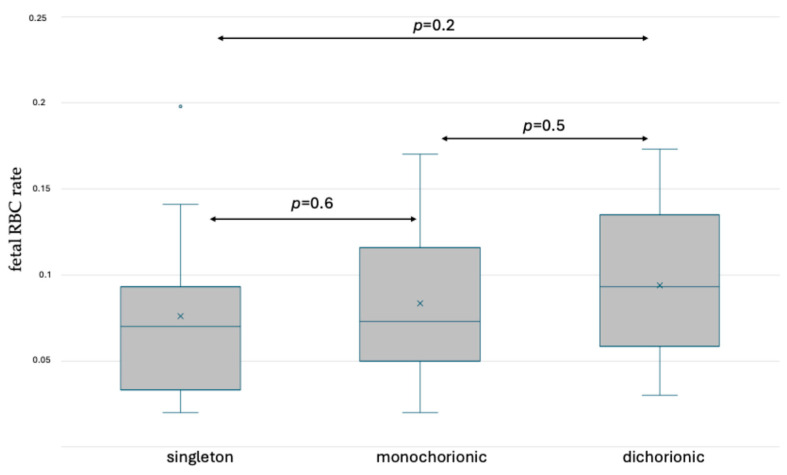
Box and whisker plots of fetal RBC ratios in singleton and twin pregnancy after delivery based on fetal hemoglobin staining. x—mean; blue line—median; blue circle—outliers.

**Figure 8:**
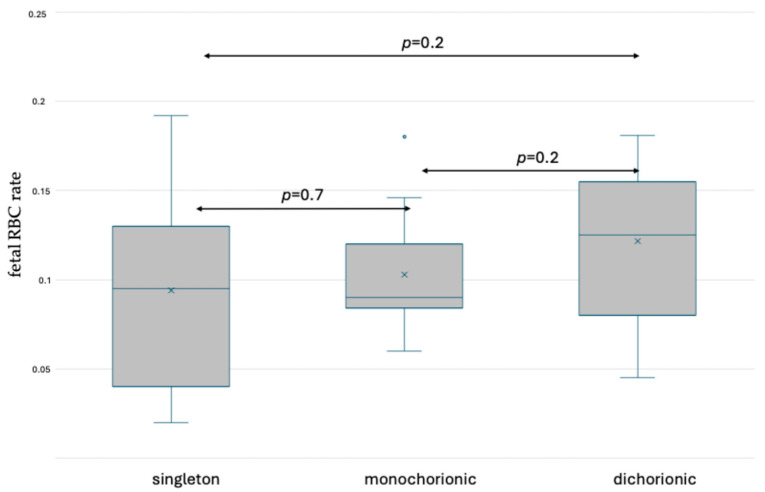
Box and whisker plots of fetal RBC ratios in singleton and twin pregnancy after delivery based on fetal hemoglobin and carbonic anhydrase staining. x—mean; blue line—median; blue circle—outliers.

The rates of fetal RBCs in the maternal circulation after delivery were assessed and the results are presented in Table S1 (Supplementary Materials). No significant differences were observed between the analyzed groups. Box and whisker plots presenting the results are shown in Figure 7 and Figure 8.

### 3.5. Comparison of F Cells Diagnosed in the Maternal Circulation before Delivery 

**Figure 9:**
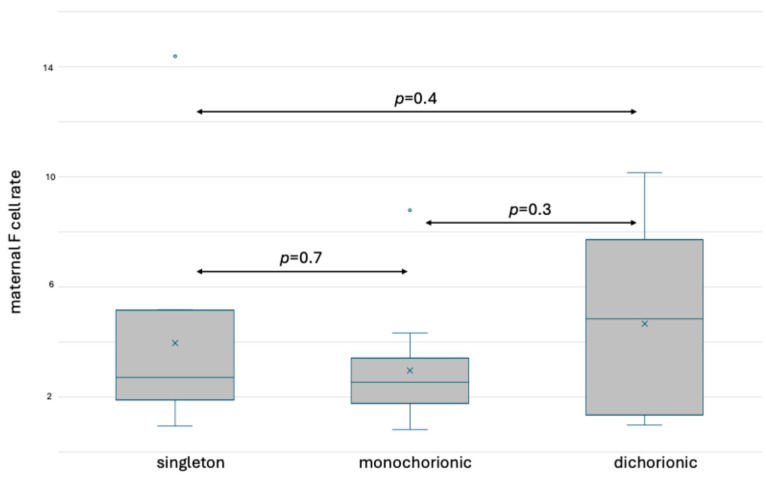
Box and whisker plots of maternal F cell ratios in singleton and twin pregnancy based on fetal hemoglobin staining. x—mean; blue line—median; blue circle—outliers.

Carbonic anhydrase and fetal hemoglobin staining allowed the separation of F cells from fetal RBCs in the maternal circulation. In HbF staining, the median rate of maternal F cells in singleton pregnancy equaled 2.72% (IQR 2.0–4.83), 2.53% in monochorionic twin pregnancy (IQR 1.77–3.36) and 4.83% in dichorionic twin pregnancy (IQR 1.49–6.97). We observed no significant differences between the groups. The results are presented in Figure 9.

**Figure 10:**
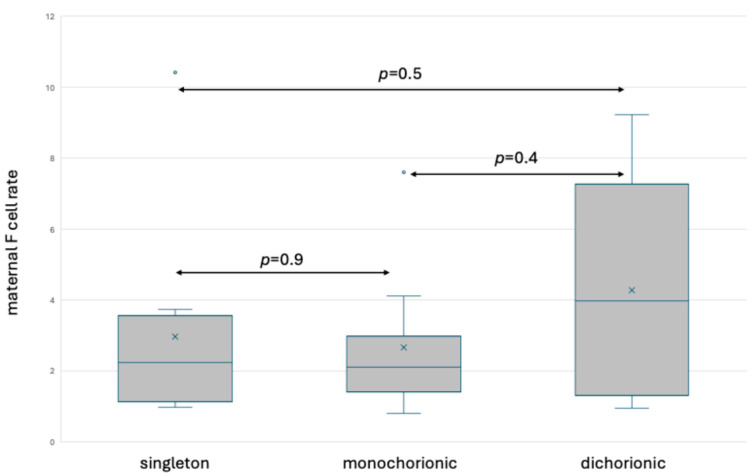
Box and whisker plots of maternal F cell ratios in singleton and twin pregnancy based on fetal hemoglobin and carbonic anhydrase staining. x—mean; blue line—median; blue circle—outliers.

The comparison of F cells between singleton and twin pregnancies using the method of simultaneous fetal hemoglobin and carbonic anhydrase staining is presented in Figure 10. The median rate of maternal F cells was 2.23% in singleton (IQR 1.3–3.93), 2.1% in monochorionic (IQR 1.51–2.98) and 3.95% in dichorionic pregnancy (IQR 1.44–6.63). No significant differences were observed depending on the methodology used.

## 4. Discussion

This is the first study presenting FMT assessment in singleton and twin pregnancies depending on chorionicity. We found that FMT was related to twin pregnancy chorionicity with the highest rate of fetal RBCs in the maternal circulation in dichorionic twin gestations both before and after delivery. An increase in fetal RBCs was observed after cesarean section in all groups. The percentage of fetal cells was low and the volume of FMT was estimated to be lower than 4 mL. 

The volume of FMT is typically low. According to Sebring and Polesky, it was below 0.025 mL in 75% of cases, below 0.5 mL in 96% and below 15 mL in over 99% [18]. The estimated volume of FMT was low in our study as well. According to the published data, the significant volume was estimated to be 30 mL, constituting the amount of fetal red blood cells covered by the standard 300 µg Rh immune globulin dose administered for the prevention of Rh sensitization [19]. However, although the 30 mL amount is significant in the prevention of Rh sensitization; it does not correlate with the risk of fetal morbidity or mortality. The threshold for a large feto-maternal hemorrhage (FMH) was established at 80 or 150 mL [20]. According to the literature, the incidence of an FMT of 30 mL was estimated to be approximately 3 per 1000 births [18,20,21,22,23]. In our study, we investigated the rate of fetal RBCs, which translated into the volume of FMT, in different groups of women, and not just the incidence of large FMH. The FMT values obtained in the studied women groups were low. Our results and the reported incidence of a large FMT put into doubt the recommended doses of anti-RhD immunoglobulin after delivery.

The FMT during delivery was investigated by other researchers, while no studies addressed the comparison of FMT between monochorionic and dichorionic twin gestations. Adeniji et al. assessed the incidence and severity of FMH in patients with singleton and twin pregnancies. They investigated 163 parturients, used the Kleihauer-Betke test to assess the FMH and found a significantly higher incidence of large FMH in twin gestations. No additional analysis of monochorionic and dichorionic twin pregnancy was available [23]. The authors concluded that twin pregnancies were associated with a higher risk of FMH. Similar results were found by David et al. The researchers investigated the incidence of FMH in 942 women and observed its incidence to be significantly higher in twin pregnancies. Again, women with twin gestations were analyzed jointly without a distinction based on chorionicity [24]. FMH was analyzed in a cohort of 313 pregnant women by Salim et al. All participants delivered by cesarean section. The authors found a similar incidence of large FMH in singleton and multiple pregnancies [25]. Moreover, several case reports presented different outcomes [2,3,12,26,27,28]. In all the abovementioned published studies, the incidence of a significant FMH was analyzed. In terms of HDF prophylaxis, not only FMH, but also FMT may be essential in alloimmunization. Sufficient prophylaxis is needed, but the adequate dose of immunoglobulin administered based on the estimated FMT could play an important role in immunoglobulin management worldwide. 

In our study, significantly larger FMT was observed in dichorionic twin pregnancies in comparison with singletons. The placenta is bigger in dichorionic twin pregnancy than in monochorionic one, and in twin pregnancy compared to singleton one [29]. We assume that the observed differences and trends in FMT are due to the placental size and the fetal-maternal interface. 

We found the highest FMT in dichorionic twin pregnancy before delivery. This means that the transfusion occurs during pregnancy and is probably related to the size of the placenta. In all analyzed groups, the rate of fetal RBCs increased after delivery. Therefore, we hypothesize that the dose of anti-D immunoglobulin in prophylaxis against HDF administered during the pregnancy should be related mostly to the kind and chorionicity of pregnancy. Our results show that the volume of FMT may be related to the size of the fetal-maternal interface and therefore is highest in dichorionic twin pregnancy. Nowadays, the dose of anti-D immunoglobulin given during gestation is the same in singleton and multiple pregnancies. Our data suggest it should be related to the multiplicity and chorionicity of pregnancy. Further research on large groups is necessary to verify this hypothesis. As establishing the correct dose of anti-D immunoglobulin based on the volume of FMT (as estimated by either Kleihauer test or flow cytometry) in every clinical case is cots ineffective, practical guidelines systematizing doses are needed. The question is—should the recommended dosed be based on gestational age, multiplicity of pregnancy or chorionicity to ensure the best and cost-effective prophylaxis?

In most adults, the range of F cells is between 0.5 and 7%. The number of these cells increases during pregnancy and their level is characterized by marked inter-individual variation, especially in pregnant women [16]. We found 2.72% (HbF staining) and 2.23% (staining fetal hemoglobin and carbonic anhydrase simultaneously) of F cells in singleton pregnancies. The rate of F cells in women with monochorionic twin pregnancy was similar (2.53% and 2.1%, respectively), and higher in women with dichorionic twin pregnancy (4.83% and 3.95%, respectively, *p* > 0.05). Inter-individual differences may be genetically determined or acquired. Corcoran et al. observed a 5-fold increase in F cells during pregnancy compared to non-pregnant populations [16]. The trend observed in our study associated with the higher rate of F cells in women with dichorionic twin pregnancy is a novel and interesting finding which needs further investigation.

The measurement of F cells and differentiation between fetal and maternal red blood cells is very difficult when using the Kleihauer-Betke method. Flow cytometry is considered as the gold standard and a reference method for FMH diagnosis [17]. It is more expensive but more accurate and automated. With this method, it is possible to precisely detect and characterize a low number of cells (e.g., fetal RBCs) present in large cell populations [30,31,32]. The evaluation of FMH using flow cytometry requires minimal blood (only approximately 20 µL for fetal hemoglobin/carbonic anhydrase staining). In contrast to the Kleihauer-Betke method, it was relatively easy to distinguish F cells among cells with adult hemoglobin A. Moreover, the use of monoclonal antibodies (e.g., anti-HbF) that we used in our study ensures high specificity and repeatability of the reaction with the antigen, making cytometric tests specific and sensitive. Due to their high specificity, immunoglobulins may be used at very low concentrations, eliminating the tendency to cross-react with other proteins and non-specific binding [33]. 

The strengths of our study include a variety of modern and precise diagnostic methods used, allowing the investigation and exclusion of maternal F cells. The quantification of FMT was established using flow cytometry which is more precise. The study groups were homogeneous and the short time between blood sample collection and the performance of the laboratory tests increase the credibility of the results. The novelty of the results brings new aspects of FMT into consideration. However, there are also limitations. The study groups are not numerous and no cases of large FMH were observed. Further studies on large groups of women with the implementation of flow cytometry FMH testing are needed to draw clinically useful conclusions.

## 5. Conclusions

FMT during pregnancy is related to the multiplicity and chorionicity of pregnancy. The obtained results suggest the necessity of further investigations on the volume of FMT during pregnancy and delivery to allow the assessment of suitable doses of anti-RhD immunoglobulin used.

## Figures and Tables

**Table 1:** Characteristics of the study group.

	Singleton Gestation *n* = 11 Median (IQR)	Twin Gestation *n* = 24 Median (IQR)	*p*	Monochorionic Twin Gestation *n* = 11 Median (IQR)	Dichorionic Twin Gestation *n* = 13 Median (IQR)	*p*
Age (years)	35 (37.3–38)	33 (29.5–35.5)	0.2	35 (30.5–37.5)	32 (29–34.3)	0.2
Maternal weight (kg)	79 (61–89)	83 (64–100)	0.3	82 (64–95)	83 (64–100)	0.9
Estimated maternal blood volume (mL)	5925 (4575–6675)	6225 (4800–7500)	0.3	6150 (4800–7125)	6225 (4800–7500)	0.9
Nulliparous *	2 (18.2)	11 (45.8)	0.1	6 (54.5)	5 (38.5)	0.7
Gestational hypertension *	1 (9.1)	0	0.3	0	0	
Gestational diabetes mellitus *	2 (18.2)	2 (8.3)	0.9	2 (18.2)	0	1
Gestational age at delivery (wks)	38 (37.3–38)	36 (36–37)	<0.01	36 (26–26)	36 (36–37)	0.8
Neonatal birth weight (g)	3140 (2920–3362)	2485 (2270–2700)	<0.01	2390 (2117–2630)	2490 (2372–2747)	0.3
Apgar score at 5 min after birth < 8	11 (100)	22 (91.7)	1	11 (100)	11 (84.6)	0.5
Neonatal hemoglobin level (g/dL)	17.9 (16.1–19.3)	17.5 (15.7–18.9)	0.4	17.4 (15.7–18.3)	17.5 (16–18.9)	0.9

* *n* (%). IQR—interquartile range.

## Data Availability

Data will be available on request.

## Supplementary Materials

The following supporting information can be downloaded at: https://www.mdpi.com/article/10.3390/jcm13123609/s1, Figure S1. An example of cytometric analysis for the detection of fetal erythrocytes in a maternal blood sample using the method for staining fetal hemoglobin. Figure S2. An example of cytometric analysis for the detection of fetal erythrocytes in a maternal blood sample using the method of the simultaneous staining for fetal hemoglobin and carbonic anhydrase. Table S1. The rates of fetal red blood cells in the maternal circulation measured by performing fetal hemoglobin staining, and fetal hemoglobin and carbonic anhydrase simultaneously.
