# Supplementary material for: Is Feto-Maternal Transfusion after Cesarean Delivery Different in Singleton and Twin Pregnancy?
Source: J Clin Med. 2024 Jun 20;13(12):3609. doi: 10.3390/jcm13123609 (PMC11204751; doi:10.3390/jcm13123609)
Supplement: Supplementary file 1 [file jcm-13-03609-s001.zip › Figure S1.pdf]

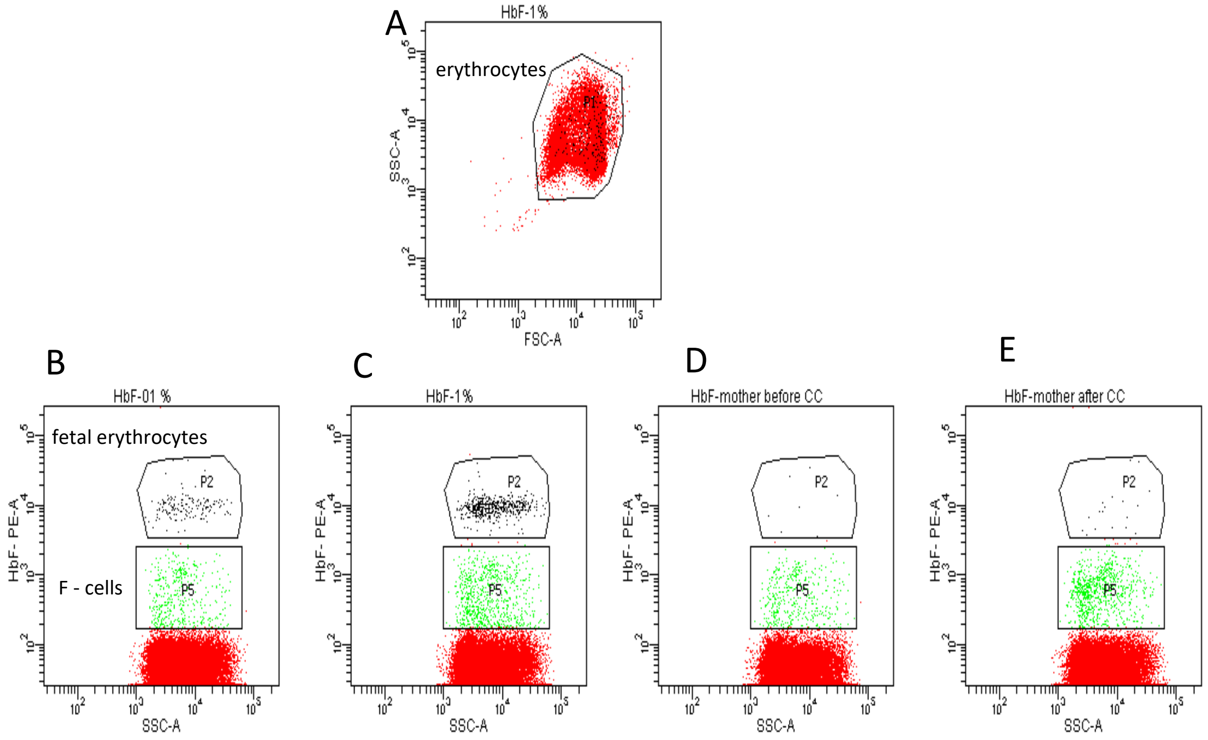

Figure S1. An example of cytometric analysis for the detection of fetal erythrocytes in a maternal blood sample using the method for staining fetal hemoglobin.

A) Erythrocyte population; B) Positive control samples of 0.1% fetal erythrocytes; C) Positive control samples of 1% fetal erythrocytes; D) Example of a sample analysis of a woman before CS; E) Example of a sample analysis of a woman after CS.
