# Supplementary material for: Is Feto-Maternal Transfusion after Cesarean Delivery Different in Singleton and Twin Pregnancy?
Source: J Clin Med. 2024 Jun 20;13(12):3609. doi: 10.3390/jcm13123609 (PMC11204751; doi:10.3390/jcm13123609)
Supplement: Supplementary file 1 [file jcm-13-03609-s001.zip › Table S1.pdf]

Table S1. The rates of fetal red blood cells in the maternal circulation measured by performing fetal hemoglobin staining, and fetal hemoglobin and carbonic anhydrase simultaneously.

Method	Singleton pregnancy	Monochorionic twin pregnancy	p	Dichorionic twin pregnancy	p*	p**
	N=11 median (IQR)	N=11 median (IQR)		N=13 median (IQR)		
Before delivery						
fetal hemoglobin staining	0.035 (0.03-0.05)	0.048 (0.027-0.089)	0.5	0.075 (0.042-0.13)	0.05	0.2
fetal hemoglobin and carbonic anhydrase staining	0.036 (0.018-0.066)	0.047 (0.033-0.103)	0.2	0.076 (0.052-0.143)	0.01	0.1
After delivery						
fetal hemoglobin staining	0.07 (0.039-0.089)	0.073 (0.052-0.115)	0.6	0.093 (0.059-0.133)	0.2	0.5
p***	0.06	0.3		0.4		
fetal hemoglobin and carbonic anhydrase staining	0.095 (0.05-0.123)	0.09 (0.084-0.12)	0.7	0.125 (0.08-0.153)	0.2	0.2
p***	0.002	0.06		0.046		

*- singleton vs dichorionic

**- monochorionic vs dichorionic

***- before delivery vs after delivery

IQR – interquartile range
